# Qualitative study on the key elements of obesity counseling in Korean Medicine

**DOI:** 10.1097/MD.0000000000023228

**Published:** 2020-11-13

**Authors:** Sungha Kim, Kyungsun Han, Jun-Hwan Lee

**Affiliations:** a Clinical Medicine Division, Korea Institute of Oriental Medicine; b Korean Medicine Life Science, University of Science & Technology, Campus of the Korea Institute of Oriental Medicine, Daejeon, Republic of Korea.

**Keywords:** counseling, Korean medicine, obesity, qualitative study

## Abstract

**Introduction:**

The increasing prevalence of obesity worldwide necessitates the provision of support for many patients. Patients with obesity appreciate receiving advice from doctors. Previous studies have qualitatively explored clinicians’ counseling for weight loss; however, this is limited to primary physicians or general practitioners working in community health centers. In contrast, Korean Medicine Doctors (KMDs) have treated patients with obesity using a holistic approach with a multicomponent approach on counseling. However, there is currently no data regarding KMDs’ consulting practices for weight loss. Therefore, the present study will explore KMDs’ experience in counseling for weight loss and describe the constituents of counseling for weight loss in Korean medicine practice.

**Methods:**

This qualitative study utilizes a phenomenological framework. The KMDs who have worked >1 year as practitioners in treating patients with obesity will be invited to describe their lived experiences of counseling patients for weight loss. Purposive and snowball sampling will be undertaken to ensure that the sample provides information-rich cases that are representative of KMDs’ experiences of counseling for weight loss. Face-to-face, individual, and semi-structured interviews will be conducted with the participants, which will be analyzed using a phenomenological method.

**Ethics and dissemination:**

Ethical approval was granted by the Human Research Ethics Committee of the Korea Institute of Oriental Medicine (I-1908/006-001). The results will be disseminated via journal articles and conference presentations.

**Trial registration number:**

Korean Clinical Trial Registry, KCT0004985.

## 1 Introduction

Obesity is a well-known risk factor for increased morbidity and mortality as well as impaired quality of life. Its prevalence has increased worldwide in the past 50 years and reached a pandemic level, as over one-third of the world's population is now classified as obese.^[1,2]^ In South Korea, the number of people with high obesity, or those which have a body mass index (BMI) of >30, is expected to increase from 3.5% in 2005 to 9.0% in 2030.^[3]^ As the BMI increases, medical costs rise progressively and are expected to grow in the next 15 years.^[4]^ Moreover, socioeconomic loss due to obesity amounted to 7.5 billion USD in 2015, which has doubled in the last 10 years in South Korea.^[3]^ Only about 15% of obese patients succeed in weight loss; however, one-third of them return to their original weight while most of them fail after 5 years.^[5]^ So far, obesity treatment strategies have not been successful in the long term.^[2]^ Hence, obesity has been recognized as a chronic disease that needs to be managed continuously over an individual's lifetime.

Obesity is more than a simple energy imbalance. Subsequently, comprehensive and multicomponent approaches that include lifestyle or behavioral training, dietary changes to reduce energy intake, and increase in physical activity have started to attract attention. Good practice can involve counseling that includes comprehensive education, lifestyle advice, and psychological support.^[4]^ For example, even the dietitian's role has transformed from being a nutrition educator to being a counsellor over the last 20 years. There has also been a concurrent shift in weight loss philosophies and practice guidelines.^[6]^ Several guidelines have been developed to assist healthcare professionals in the treatment of obesity and promote effective counseling.^[7,8]^ Doctors use these guidelines as basis for outlining counseling strategies for obesity which include lifestyle changes, dietary modification, and increased physical activity.^[4]^ In a way, counseling is the key means to stabilize the patients’ psychological urge to overeat, correct their knowledge on weight loss, and eventually balance their input and output of energy.

Previous studies have demonstrated the effectiveness of counseling for weight loss (i.e., counseling itself, or counseling combined with medicine).^[9–12]^ Using qualitative study, they have explored the clinicians’ approach to weight counseling, including what advice to provide during a routine consultation.^[13–18]^ So far, the interviewees of the previous studies have been limited to primary physicians or general practitioners working in community health centers. Accordingly, they focused on how doctors raise the topic of obesity with patients who come to their clinics for other reasons. In those situations, counseling for weight loss is easily overlooked and doctors tended to have negative attitudes towards it.^[14–16,18]^

Korean Medicine (KM), a conventional health care covered by the national medical healthcare system, are playing an important role in the National Health Service of South Korea especially for obese patients.^[19–21]^ Since previous studies in KM have confirmed its effectiveness, KM therapies such as acupuncture, herbal medicine, and counseling are frequently used to treat obesity.^[22–24]^ Especially, counseling is considered as an essential practice that improves the patient's understanding and participation, and leads to the patient's behavioral modification. A manual for standard counseling for obese patients in KM was developed by recognizing counseling as an important factor in the treatment of obesity.^[8]^ The manual covers pattern identification, and Sasang constitution to classify patients into 6 patterns and 4 constitution respectively to prescribe herbs or manage prognosis factors like abdominal obesity. However, there is currently no data on the consulting practices of Korean Medicine Doctors (KMDs) related to weight loss.

Phenomenology is a philosophical approach used in describing the internal meaning and structure of experience.^[25]^ Thus, this approach is useful in understanding the essential meaning of counseling in KM practice for treating patients with obesity, and researching the essential structure of human experience.^[26]^ In applying this, researchers will use the qualitative method to describe the KMDs’ counseling for weight loss and the constituents of counseling for weight loss in KM practice.

The study aims to explore the experiences of KMDs when they advise about weight loss. Specifically, this study aims to investigate the following research questions: What are the experiences of KMDs while they provide counseling for weight loss? How do KMDs decide what to say to their patients for their weight? What are the KMDs’ perceptions and attitudes towards counseling in the management of obesity? What are the objectives and roles of counseling in the management of obesity? What advice do KMDs provide during counseling for weight loss? What do KMDs say to their patients about their weight? What is the difference between the KMD's counseling and obesity counseling by other experts, such as dietitians or personal trainers? As individual treatment is emphasized in the management of obesity, what are the individual strategies in counseling?

## 2 Methods and analysis

### 2.1 Qualitative study design

This research will employ a qualitative design to explore the experiences of KMDs in counseling for weight loss. We chose the qualitative descriptive approach because it provides broad and rich information and straightforward descriptions of the participants’ perspectives on counseling for weight loss. Moreover, we chose this approach for its usefulness in gaining a preliminary insight into a topic and collecting the first-hand experiences of participants. Lastly, the conduct and reporting of this study will be in accordance with the Consolidated Criteria for Reporting Qualitative Research checklist for interviews to ensure rigor, comprehensiveness, and credibility.^[27]^

This study will be situated within a phenomenological framework as we are attempting to understand the experiences of KMDs on counseling for weight loss.^[28,29]^ Moreover, we used the phenomenological framework since it requires the researchers to examine the issue by collecting data from individuals who have first-hand experience. According to Husserl and Moran,^[30]^ the best way to study a phenomenon is to go back to “the things themselves.” Therefore, we will go back to the KMDs to gain a valid understanding of their personal experiences and perceptions of counseling in the treatment of obesity.

### 2.2 Study sample and recruitment

Researchers will interview up to 15 KMDs who have experienced counseling for weight loss, and recruit them using purposive or snowball sampling. Purposive sampling is the method in which a researcher intentionally approaches participants who are suitable for research and selects them.^[31]^ Since creating specialized obesity clinics that focus on obesity management has become a trend, the number of hospitals operating obesity clinics has increased rapidly,^[32]^ with >3000 obesity clinics in Seoul alone.^[33]^ Because of this, the Society of Korean Medicine for Obesity Research (SKMOR), with a membership of >500 KMDs who have worked in specialized obesity clinics, was established. Hence, researchers will contact the SKMOR to look for qualified people who are interested to be participants in our study. One of the researchers (KH) is an editorial director of the SKMOR, and she will have participant contact. On the other hand, snowball sampling is the method in which participants introduce applicants to the researcher,^[34]^ where suitable applicants are registered as participants.

The recruitment of the research participants, the interviews, and the analysis of the interviews will be performed at the same time. When we have repeated the interviews and analyses, there will be a time when no new themes are being generated, which is called “data saturation.”^[35]^ When data saturation is reached, researchers will stop recruiting participants.

The potential participants will be provided with information about the aims and methods of the study and be assured that their confidentiality and privacy will be maintained. The potential volunteer recruits will also be informed that they are free to withdraw from the study at any time. Those agreeable to participating will provide written and informed consent.

The inclusion criteria will include doctors who finished their 4- or 6-year course in a nationally accredited KM university, have a KMD license certified by the Ministry of Health and Welfare in South Korea, and have experience as practitioners of medical management for weight loss for >1 year. The exclusion criteria will be those who did not agree to participate and considered to be inappropriate by the researchers (i.e., counseling time is <5 minutes).

### 2.3 Data collection and recording

This study will be conducted from September 2019 to October 2020. Two researchers with experience in managing patients with obesity and qualitative research will conduct the interviews. The main method of data collection will be in-depth interviews that use open questions and semi-structured follow-up interviews (Table 1). Examples of questions for the KMDs include “What do you say to patients about their weight?” “What is the priority in the management of obesity?” “What do you advise about weight loss?” “What are the objectives and the role of counseling in the management of obesity?” “How important is counseling for the outcomes and prognosis of obesity?” “What is the difference between KMD's counseling and obesity counseling by other experts, such as dietitians or personal trainers?” “During the management of obesity, individual treatment is emphasized; what are the individual strategies in counseling?” and “Is standardized counseling available according to the guidelines about consultation for obesity?”

**Table 1:** Interview guide.

Topic	Main question	Probe
Introduction	Interviewers will state the purpose and notice of this interview to interviewees and create a comfortable atmosphere prior to the full-scale theme and aim to identify the interviewee's profileGreetings, words of appreciation, and reminders (e.g., to turn off the mobile phones)Introduction of researchersExplanation of the purpose of the interviewExplanation of how to proceed	
Warm-up questions (demographic characteristics)	Overview of KM treatment for obesity“Please tell us about the counselling for patients with obesity currently being done in KM clinics.”Status of obesity care, number of patients, consultation period, etc.“How important is counselling in treatment outcomes and prognosis of weight loss?”	
Main questions	Core contents of KM counseling for weight loss“What is the purpose of counselling?”“What should be the first approach in counselling?”“Compared to other obesity specialists such as dietitians and exercise therapists, what makes KM counselling different?”“How important is pattern identification in counselling for weight loss?”“How important is Sasang constitution is in counselling for weight loss?”	Personal experience
Additional questions	Questions about the manual for standard counseling for patients with obesity in KM“What is the purpose of the manual?”“In what areas would it be helpful to use the manual?”“How would you like the manual to be improved to reflect the characteristics of KM counselling?”“How could the manual be improved to reflect the reality of the treatment?”	
Ending	Future direction of counseling for weight loss in KM1. “What if the National Health Insurance covers KM standard counselling?”2. How would it impact the KM industry and the nation if all the KMDs had standardized counselling?”	

The interviews will be held with consideration to the doctors’ schedules in a quiet and comfortable place. To eliminate distractions, mobile phones will be turned off and access to the location will be limited. The length of each interview is expected to range from 30 to 120 minutes, and each participant, who will be interviewed once or twice, shall be given a small compensation of approximately 40 USD thereafter. The interviewers will write field notes describing the setting and experience during each interview. Moreover, researchers will observe bodily expressions. Audio will be recorded, transcribed verbatim, and saved to a computer immediately after the interview upon the participant's consent. Additionally, researchers will ensure authenticity by utilizing field notes. Lastly, data collection will be discontinued when the theoretical saturation is reached; that is when data are duplicated.^[35]^

All participants will be contacted by e-mail or telephone and asked if they agree to be interviewed. Subsequently, a face-to-face interview will be conducted at their preferred location after obtaining consent.

### 2.4 Data analysis

The study aims to analyze the experiences of KMDs in order to generate visible essential meanings attached to counseling for weight loss. To do this at a more structural level than in the original descriptions, our analysis will be inspired by the work of Giorgi,^[36]^ who developed the phenomenological method for analyzing empirical research data, to facilitate the description of the essential meaning structure of the phenomenon being studied. Researchers will read all of the data, determine the meaning of the units of each interview, mark each shift in meaning by using a keyword related to the units, and organize each interview with respect to the meaning of the units which will yield a sketch of the preliminary constituents, consecutively. Thereafter, researchers will transform the meaning of the units into condensed descriptions and read all of them to synthesize the constituents and identify the essential meaning of the descriptions.^[37]^ Data analysis will be conducted by the first author (SK) and assessed by the second author (KH). Disagreements will be resolved by discussion.

### 2.5 Ethics and dissemination

Ethical approval has been granted by the Korea Institute of Oriental Medicine's Research Ethics Committee (I-1908/006–001). Informed consent will be obtained from all participants and they will be guaranteed confidentiality and anonymity. Consent to participate will also be obtained via written consent. Following the analysis, the findings will be submitted for publication in peer-reviewed journals. They will also be presented at relevant international academic conferences in the areas of obesity or counseling.

## 3 Discussion

This study will provide information on the experiences of KMDs in counseling for weight loss. The qualitative nature of this study will provide an in-depth understanding of the perspectives and experiences of KMDs on counseling for weight loss, especially the roles and objectives of counseling. The study will extend the conceptual understanding of the experiences of KMDs as regards counseling for weight loss.

The advice of the KMDs will provide practitioners information on how to perform better counseling, offer tips on how to adapt the guidelines, and provide a greater understanding of the roles of counseling in the management of obesity. Besides, one of the characteristics of KM is the pattern identification and Sasang constitution.^[38,39]^ KMDs use these to categorize patients with obesity for prescribing herbs or managing prognosis factors like abdominal obesity. Patients with obesity are categorized into 6 patterns: spleen deficiency, yang deficiency, indigestion, stagnation of the liver Qi, blood stasis, and phlegm. For example, a patient diagnosed as stagnation of the liver Qi, the key factor of counseling is to teach how to handle stress. A patient diagnosed as yang deficiency is easy to gain weight, so lowering the target weight is essential. With this study, we can figure out how pattern identification and Sasang constitution are being used in counseling for weight loss in KM.

The findings of this study may lead to follow-up surveys on counseling for weight loss in South Korea, and future research to revise the manual for standard counseling for patients with obesity in KM. Further, these findings may contribute to policy recommendations and legislation so that counseling can be covered by the National Health Insurance system in South Korea.

## Author contributions

**Conceptualization:** Sungha Kim.

**Formal analysis:** Sungha Kim.

**Investigation:** Sungha Kim, Kyungsun Han.

**Methodology:** Sungha Kim, Kyungsun Han.

**Supervision:** Sungha Kim.

**Writing – original draft:** Sungha Kim.

**Writing – review & editing:** Kyungsun Han, Jun-hwan Lee.
